# Study on a New Environmentally Friendly Synthetic Fluid for Preparing Synthetic-Based Drilling Fluid

**DOI:** 10.3389/fchem.2020.539690

**Published:** 2020-12-14

**Authors:** Yuxue Sun, Xiuyu Zhu, Jingyuan Zhao, Dianjie Sui

**Affiliations:** ^1^College of Petroleum Engineering, Northeast Petroleum University, Daqing, China; ^2^Drilling Engineering Technology Research Institute, Daqing Drilling Engineering Company, Daqing, China; ^3^College of Mechanical and Electrical Engineering, Guangdong University of Petrochemical Technology, Maoming, China

**Keywords:** hydrogenation technology, performance evaluation, synthetic fluid, environmentally friendly, synthetic-based drilling fluid

## Abstract

In view of the pollution problems related to using oil-based drilling fluids, we prepared a new environmentally friendly synthetic fluid, namely, NSF, for use in synthetic-based drilling fluid instead. We used heavy hydrocarbons from Daqing as raw material in our preparation and employed few-steps refining techniques, such as hydrodesulfurization, hydrodearomatization, and hydrogenation. After the treatment, the sulfur and aromatic hydrocarbon contents in the NSF were <0.5 mg/L, meeting international environmental standards. NSF is composed mainly of C_12_-C_22_ hydrocarbons, and is characterized by low impurity content, small specific gravity, and easy degradation. In addition, the LC50 value is >1,000,000 mg/L water-soluble fraction; therefore, its toxicity is <10% of that of diesel in oil-based drilling fluid and, as the flash point of NSF is about 134°C, the related fire hazard is low. The synthetic-based drilling fluid with NSF has the advantage of causing no swelling damage to the reservoir, and saves the cost of drilling fluid because it doesn't need to deal with the environmental problems (the sulfur and aromatic hydrocarbon contents were <0.5 mg/L) and field problems caused by the reaction between drilling fluid and reservoir, and greatly increases the ROP through reducing the drill pipe sticking and with C_12_-C_22_ hydrocarbons in synthetic-based fluid, and safely uses, and has stable drilling fluid performances which are flash point (134°C) and low fire hazard. We have therefore achieved our aim of preparing a high-quality and non-toxic synthetic fluid, of which the aromatic hydrocarbon and sulfur contents meet international standards. Using this synthetic-based drilling fluid therefore facilitates environment protection drilling.

## Introduction

The drilling fluids that commonly used in the oil field can be divided into water-based drilling fluid, oil-based drilling fluid and synthetic-based drilling fluid according to their properties. Water-based drilling fluid is one of the earliest used systems in drilling. And with the increasing complexity of drilling conditions, water-based drilling fluids with high temperature resistance, high density, and strong inhibitor are gradually studied, such as using nanomaterials to replace KCl and produce a new kind of inhibitor which can reduce drilling and disposal cost. Also, some researchers reported a novel kind of water-soluble acrylic acid grafted activated carbon and Cetyltrimethylammonium modified grapheme which can also reduce water invasion and protect the clay from water. However, for drilling long sections in wellbore, it still can cost much and cause environmental problems. Therefore, it is always a problem to find biogradable materials and nanomaterials which are good for environment and reservoir (Saleh and Ibrahim, 2019; Ibrahim and Saleh, 2020; Rana et al., 2020).

What's more, oil-based drilling fluid that is used to replace the water-based drilling fluid has good performance, it can cost much, about as three times as water-based drilling fluid, and, its use remains limited because of the attendant pollution problems. However, in recent years, the use of gas-based oil instead of ordinary diesel as base oil has alleviated the pollution problems, but the production process is complex and expensive. Moreover, natural gas is scarce in China; consequently, using this type of base oil is not suitable for our country (Yan, 2001; Perry and Lee, 2007; Hao, 2015; Xie, 2017). In order to meet the environmental requirements, synthetic drilling fluid which can replace traditional oil-based drilling fluid is increasingly popular and widely used in fields. Synthetic based drilling fluid regards synthetic fluid as continuous phase and brine or water as dispersed phase. Moreover, the synthetic-based fluid is a relatively pure material without low molecular weight components. Therefore, it is less toxic and can be biodegraded under aerobic and anaerobic conditions, which is less harmful during fluid treatment. The performance of synthetic drilling fluid is similar to that of oil-based drilling fluid, but it is safe to use and has little environmental pollution (Candler et al., 1993; Patel, 1998).

Therefore, the aim of this study was to prepare a low-cost, high-quality, non-toxic synthetic fluid, namely, NSF, of which the aromatic hydrocarbon and sulfur contents meet international standards. We employed hydrogenation and catalytic oxidation technology and used heavy hydrocarbons from Daqing to prepare this new synthetic fluid and synthetic-based drilling fluid that is not only environmentally friendly but also has strong practicability.

## Materials and Methods

### Preparation of Materials and Process Equipment

#### Materials

The physicochemical index of the heavy hydrocarbons we used as raw material are (1) a distillation range of 80–280°C, (2) condensation point of−20 to−40°C, (3) flash point of 30–50°C, (4) sulfur content of 250 mg/L, (5) aromatic hydrocarbon content of 6–8%, and (6) alkene content of 2–3%.

#### Equipment

**Figure 1:**
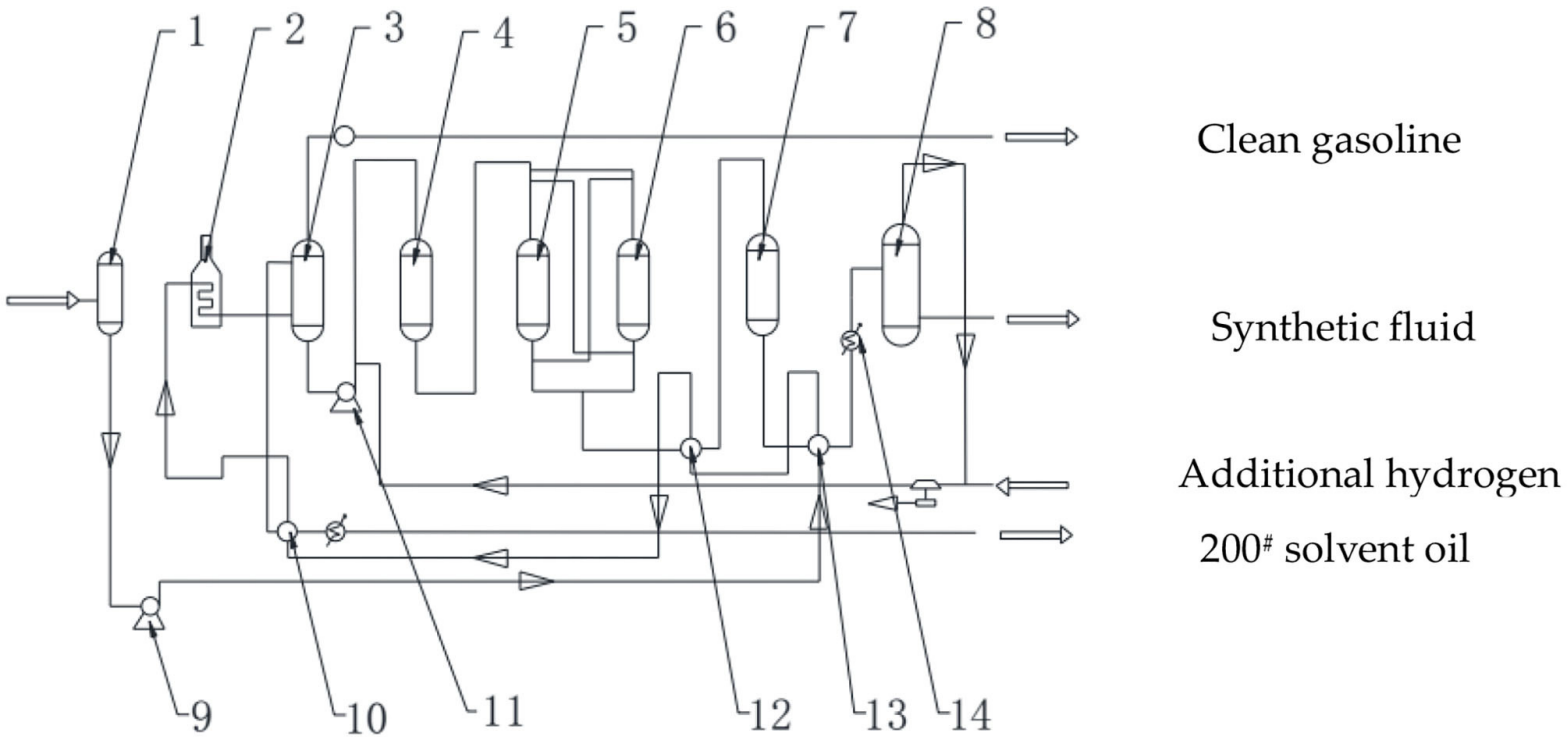
Process flow diagram of NSF synthetic fluid preparation.

As shown in Figure 1, the equipment included (1) a stock cushion tank, (2) heating furnace, (3) fractionating tower, (4) first-order hydrogenation reactor, (5) mesothermal temperature zinc oxide sweetener A, (6) mesothermal temperature zinc oxide sweetener B, (7) second-order hydrogenation reactor, (8) cooling extractor, (9) stock hydrocarbons pump, (10) third heat exchanger, (11) supercharged pump, (12) second heat exchanger, (13) first heat exchanger, and (14) a condenser.

#### Process

After fractionation, hydrogenation, desulfurization and aromatization, cooling, and other processes, the aromatic content of the final synthetic fluid was <0.5 mg/L and the sulfur content was also <0.5 mg/L. This means that the quality of this fluid was consistent with the accepted standards. The main processes are as follows.

(1) Fractionation

The heavy hydrocarbons in the stock cushion tank (1) is transported by the stock hydrocarbons pump (9), in turn, with the second hydrogenation-generated fluid of the second-order hydrogenation reactor (7) to exchange the heat in the first heat exchanger (13) and, subsequently, into the second heat exchanger (12) to exchange the heat with the generated fluid (220°C). After mesothermal temperature zinc oxide fine desulfurization (220°C), it is transported into the third heat exchanger (10) to exchange the heat with the solvent oil (200^#^ naphtha), which is separated from the fractionator (3). Finally, the agent fluid is heated to the separation temperature (200–260°C) with the heating furnace (2) and transported into the fractionator (3). After the agent fluid is separated from the fractionator (3), the clean gasoline is obtained at the top of the fractionating tower and the 200^#^ naphtha is measured in the tower. The bottom fluid of the fractionating tower is transported to the first hydrogenation reactor (4) at a pressure of 0.5 MPa, with the supercharged pump (11).

(2) First hydrogenation

The additional hydrogen supplied by the compression device is mixed with the bottom fluid before being pumped by the supercharged pump (11), and the mixture of bottom fluid and hydrogen enters the first-order hydrogenation reactor (4) from the upper part of the tower. In this process, the mixture of bottom fluid and hydrogen is always in the condition of 200–260°C and 0.5 MPa. Next, the hydrogenation reaction is conducted at a temperature of 200–220°C, pressure of 0.5 MPa, and airspeed of 1 h^−1^, under the existence of catalyst, the sulfide in oil reacts with hydrogen, and after once of this process, the organic sulfur at the bottom of the tower is converted into H_2_S, and most of the alkenes are converted into alkanes. The amount of this process is depend on if the sulfur content meets the demand of standard which is 0.5 mg/L, if not, repeat the process again. The first-order hydrogenated synthetic fluid is discharged from the bottom of the first-order hydrogenation reactor (4) into the zinc oxide desulfurization sweetener.

(3) Zinc oxide desulfurization

The H_2_S from the first-order hydrogenated synthetic fluid is then reduced to <0.5 mg/L in the zinc oxide sweetener through the reaction between H_2_S and ZnO, the temperature is controlled at 200–220°C and the pressure is 0.5 MPa. After this period, and the generated synthetic fluid after desulfurization is transported into the second hydrogenation reactor (7) under 200–220°C and 0.5 MPa.

(4) Second hydrogenation

The hydrodearomatization is conducted at a temperature of 120–160°C, pressure of 0.5 Mpa, and airspeed of 1 h^−1^ in the second-order hydrogenation reactor (7), the arene reacts with hydrogen and is converted into alkane and cycloalkane. Subsequently, after second-order hydrogenation, the generated synthetic fluid and excess hydrogen are discharged from the bottom of the second-order hydrogenation reactor (7) to the first heat exchanger (13).

(5) Cooling and separation

The second-order hydrotreated synthetic fluid enters the condenser (14) after heat exchange in the first heat exchanger (13) with the stock heavy hydrocarbons and, subsequently, enters the cooling separator (8) after cooling at the condenser (14). The final product is obtained after gas–liquid separation in the cooling separator (8). The final product is transported to the product cushion tank if the synthetic fluid product satisfies the demand of both aromatics and sulfur content being <0.5 mg/L. The fluid in the product tank is the synthetic fluid of the environmentally friendly drilling fluid. If the content of aromatics and sulfur are not below the standard condition (0.5 mg/L), repeat the above process. The hydrogen is compressed by the circulating hydrogen compressor after mixing the circulating hydrogen from the cooling separator (8) and the supplemental hydrogen and, subsequently, it is transported to be mixed with the raw material or to be used as cold hydrogen.

### Desulfurization and Dearomatization Experiment

In this experiment, the sulfur content in the raw material was sorted into thiol, sulfur ether, and sulfur dioxide, which have high aromatics content, as shown in Table 1.

**Table 1:** Nature of hydrocarbons in the laboratories.

**Number**	**Sulfur content W/%**	**Sulfur form/mg/L**				**Hydrocarbon series component/%**		
		**Thiol**	**Sulfur dioxide**	**Sulfur ether**	**Others**	**Aromatic hydrocarbon**	**Satisfied hydrocarbon**	**Non-hydrocarbon**
1	0.88	3,900	1,228	3,260	408	25.1	74.7	—
2	0.74	2,821	2,181	2,019	216	23.0	77.0	—
3	1.69	9,970	4,260	2,790	—	19.6	80.1	0.3
4	1.20	—	—	—	—	22.4	77.4	—
5	1.90	14,380	—	—	—	21.1	78.6	—

Thiol and sulfur dioxide are both active sulfides with unpleasant odor, are significantly corrosive to Cu metal, and can reduce the stability of oil products. Sulfur ether is inactive sulfide, but it is also corrosive. The sulfur oxides produced by the combustion of these three sulfurs cause environmental pollution. Furthermore, if the aromatic content is too high, the drilling fluid will be highly toxic and will cause serious pollution. The international standards for sulfur and aromatics contents are shown in Tables 2, 3, and the classification criteria for toxicity are shown in Table 4.

**Table 2:** The new criterions of hydrocarbons.

**Countries**		**Sulfur content/%**	**Aromatic hydrocarbon content/%**	**Cetane number content/%**
America		≤ 0.05	≤ 20	≥40
Sweden	I	≤ 0.001	≤ 5	
	II	≤ 0.005	≤ 10	≥50
	III	≤ 0.050	≤ 20	≥50
Denmark		≤ 0.050		≥50
Japan		≤ 0.050		≥45
Korea		≤ 0.050		

**Table 3:** The international association of oil and gas production for the classification of drilling fluid system.

**Classification**	**Characters**	**Palycyclic aromatic content/%**	**Generalized case aromatic hydrocarbon content/%**	**Categories**
I	High aromatic content	>0.35%	2.4–25%	Diesel and traditional mineral oil
II	Medium aromatic content	0.001–0.35%	0.5–5%	Low aromatic hydrocarbon mineral oil
III	Low aromatic content	<0.001%	<0.5%	Synthetic or highly refined mineral oil

**Table 4:** The classification of toxicity.

**Categories**	**Poisonousness quality concentration value 96 h LC50 value/(mg·l^**−1**^)**
Non-toxic	>10,000
Slightly toxic	10,000
Medium toxic	100–1,000
Toxic	1–100
Significantly toxic	<1

We compared the synthetic fluid of the drilling fluid required particularly for the exploitation of shale formations with the above criteria, finding that the conventional raw material has both high sulfur and high aromatic contents. Therefore, to improve the material to be used as synthetic fluid in drilling fluid, it needed to be desulfurized and dearomatized.

#### Desulfurization Experiment

Catalytic cracking was used in this experiment, as it can convert the organic sulfur compounds in the stock hydrocarbons into hydrogen sulfide, which escapes in the form of gas. Subsequently, the performance of the stock hydrocarbons could be improved by catalytic oxidation, after it meets the above standards (Wu, 2000; Zhang et al., 2018).

##### Experimental Principles

**Figure 2:**
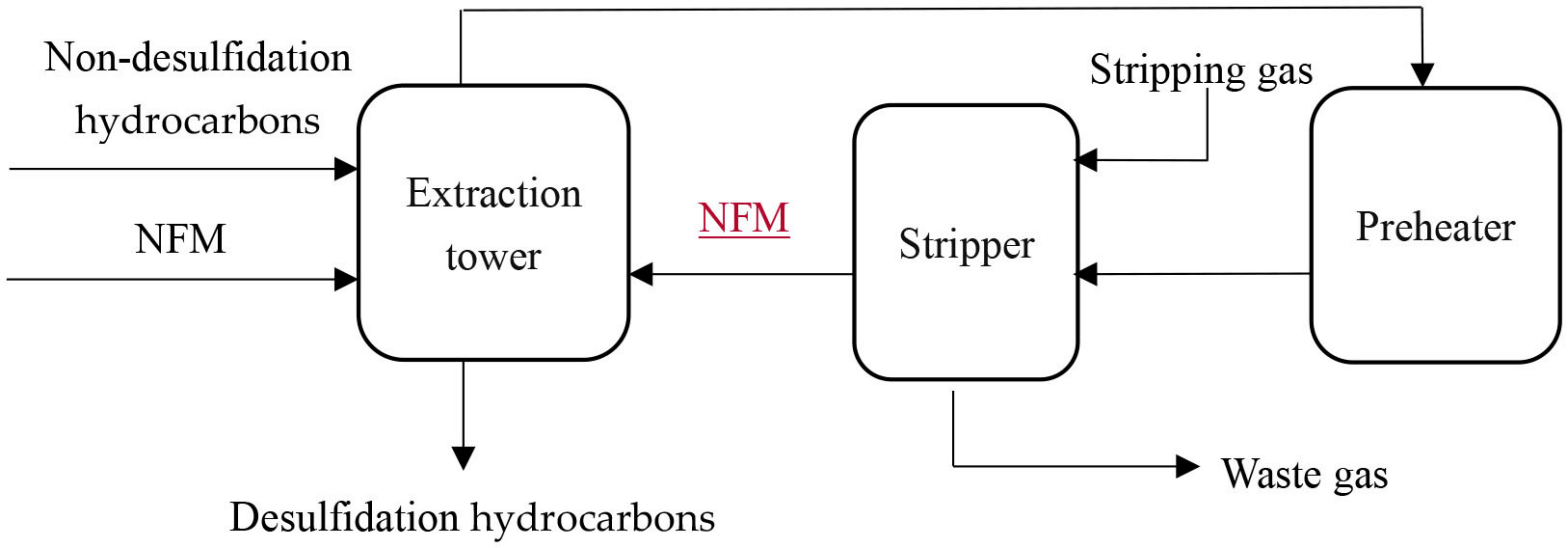
Desulfurization process flow diagram.

Catalytic cracking desulfurization is essentially the carbon-sulfur bond in the organic sulfide molecule being broken by the catalyst (acidic condition) to form hydrogen sulfide, changing the original form of the sulfur, after which it is removed (Liao et al., 1999; Parkinson, 2000). The stock hydrocarbons forms gas, synthetic fluid, oil, and coke during the catalytic cracking, and some coke is deposited onto the surface of the catalyst. As the carbon deposition increases, the activity of the catalyst decreases, eventually leading to a decrease in the desulfurization rate. At this point, regeneration of the catalyst is carried out, meaning that the original reaction is stopped, and the steam mixture is used to burn the carbon deposit, thereby restoring the activity of the catalyst (Wang et al., 2009; Halbert and Greeley, 2000). The relevant equations are as follows and the process flow diagram is shown in Figure 2.

(1) $$\begin{array}{l} \text{R}-\text{SH}\to {\text{R}}^{+}+{\text{H}}_{2}\text{S} \end{array}$$

(2) $$\begin{array}{l} \text{R}-\text{S}-\text{R}\to \{\begin{array}{c} {\text{R}}^{+}+\text{RSH}\\ \text{2}{\text{R}}^{+}+{\text{H}}_{2}\text{S} \end{array} \end{array}$$

##### Experimental Conditions

The reaction conditions of the experiments were controlled, and the experiments were conducted at airspeeds of 1.0, 2.0, and 3.0 h^−1^, respectively. Each group of airspeeds was tested at different temperatures, namely, 380, 430, 480, and 530°C, as shown in Table 5.

**Table 5:** Hydrocarbons and catalyst desulfurization experiment.

**Airspeed**	**380**^****°****^**C**			**430**^****°****^**C**			**480**^****°****^**C**			**530**^****°****^**C**		
	**Number**	**S/%**	**W/%**	**Number**	**S/%**	**W/%**	**Number**	**S/%**	**W/%**	**Number**	**S/%**	**W/%**
1.0	G-1-1	0.09		G-2-1	0.09		G-3-1	0.08		G-4-1	0.05	
	G-1-4	0.13		G-2-4	0.09		G-3-4	0.06		G-4-4	0.05	
	G-1-6	0.15		G-2-6	0.11		G-3-6	0.06		G-4-6	0.04	
	G-1	0.10	97.30	G-2	0.07	96.80	G-3	0.07	88.90	G-4	0.03	83.20
2.0	G-5-1	0.10		G-6-1	0.10		G-7-1	0.06		G-8-1	0.06	
	G-5-4	0.12		G-6-4	0.10		G-7-4	0.07		G-8-4	0.07	
	G-5-6	0.12		G-6-6	0.10		G-7-6	0.08		G-8-6	0.09	
	G-5	0.11	97.90	G-6	0.10	96.90	G-7	0.07	95.90	G-8	0.05	90.80
3.0	G-9-1	0.16		G-10-1	0.13		G-11-1	0.11		G-12-1	0.06	
	G-9-4	0.15		G-10-4	0.12		G-11-4	0.10		G-12-4	0.08	
	G-9-6	0.17		G-10-6	0.13		G-11-6	0.08		G-12-6	0.08	
	G-9	0.16	97.70	G-10	0.11	97.20	G-11	0.09	94.20	G-12	0.05	90.80

*S%, the content of sulfur in desulfurization hydrocarbons (weight %); W%, desulfurization hydrocarbons weight yield*.

As Table 5 shows, at a certain airspeed, the sulfur content of the desulfurized fluid decreases as the temperature increases, with the fluid yield decreasing as well. In this instance, the fluid yield was not ideal and, to improve the desulfurization effect, the procedure would have to be conducted at a higher temperature and lower airspeed. Therefore, the conditions we chose were 2.0 h^−1^ and a temperature of 480°C.

##### Experimental Results

The properties of the stock hydrocarbons after catalytic desulfurization are shown in Table 6, which indicates that the cracking reaction occurs simultaneously with desulfurization.

**Table 6:** Properties of raw material after desulfurization.

**Number**	**Temperature of reaction/^**°**^C**	**Sulfur content W/%**		**Desulfurization rate/%**	**Alkene/%**
		**Stock hydrocarbons**	**After desulfurization**		
1	530	1.68	0.50	70.00	7.60
2	530	1.18	0.38	67.60	4.00
3	480	1.44	0.55	61.70	4.80
4	490	1.91	0.95	50.50	—
5	440	1.91	1.12	41.20	—

As Table 6 shows, at a certain airspeed, the desulfurization rate increases as the temperature increases, and the higher temperature, the lower the gap of desulfurization rate.

After the catalytic desulfurization, the sulfur content of the stock hydrocarbons remained high; therefore, further experiments were conducted employing catalytic oxidation to remove more thiols from the sulfur. The catalytic oxidation experiments require two procedures, namely, extraction and deodorization (Basu et al., 1993; Ji, 2013; Xie, 2014). In the extraction experiment, the catalyst was dissolved in the lye and mixed with the hydrocarbons. The thiol reacted to form sodium thiolate and separated from the hydrocarbons and, subsequently, air was added to oxidize the sodium thiolate to form a disulfide precipitate, which was then separated. The relevant equations are as follows:

(3) $$\begin{array}{l} \text{RSH}+\text{NaOH}\to \text{NaSR}+{\text{H}}_{2}\text{O} \end{array}$$

(4) $$\begin{array}{l} 2\text{NaOH}+{\text{H}}_{2}\text{S}\to \text{N}{\text{a}}_{2}\text{S}+2{\text{H}}_{2}\text{O} \end{array}$$

(5) $$\begin{array}{l} 4\text{SRNa}+{\text{O}}_{2}+2{\text{H}}_{2}\text{O}\to 2\text{RSSR}+4\text{NaOH} \end{array}$$

**Figure 3:**
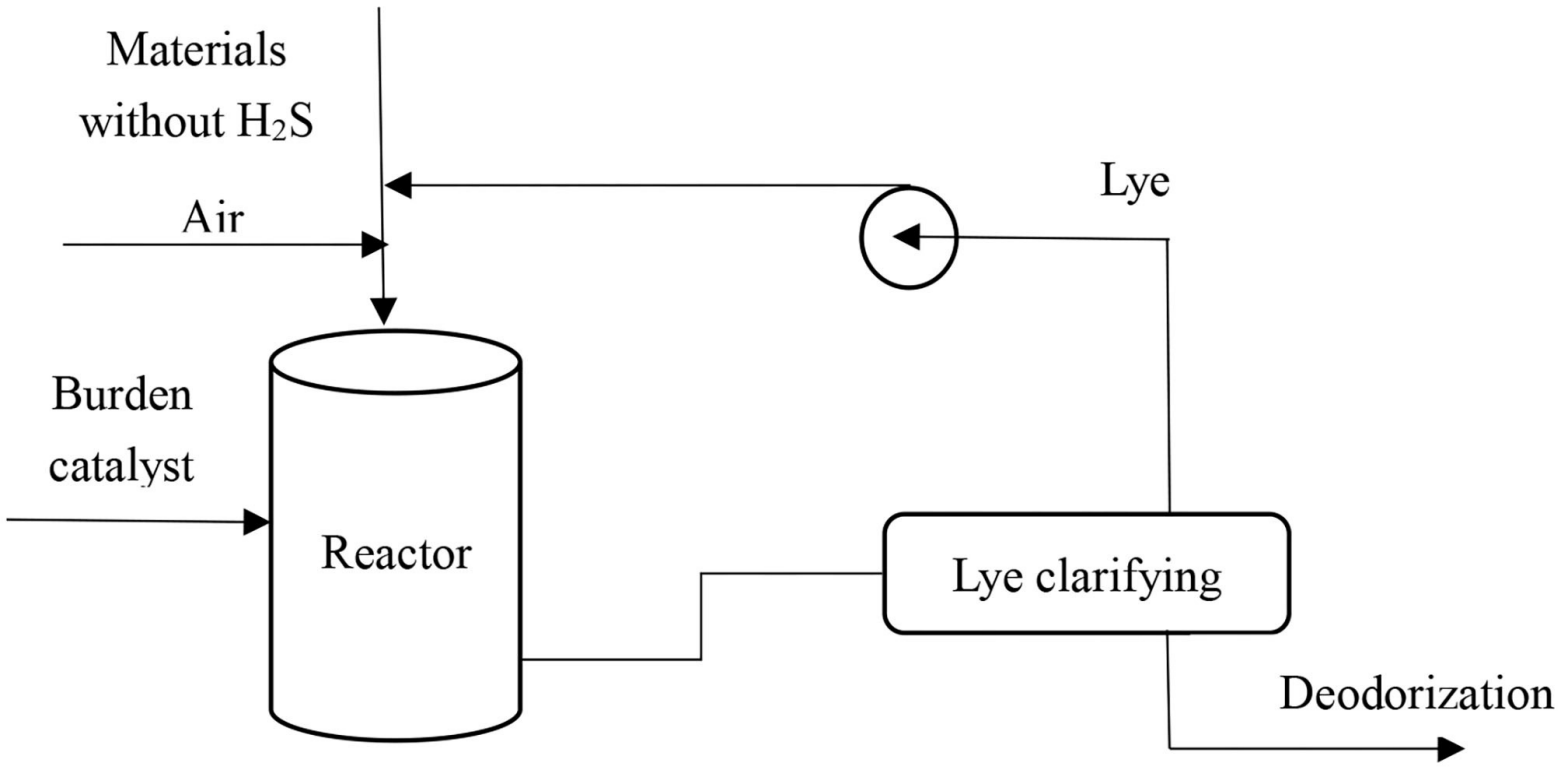
Conventional deodorization process under atmospheric pressure and 25°C.

Afterward, deodorization was carried out, with the thiol converted into disulfide, whereas the total sulfur content remained unchanged. The thiol was removed after being extracted and deodorized. In this experiment, sulfonated asphalt was used as the catalyst under atmosphere pressure and at the temperature of 25°C. The relevant equations are as follows and the deodorization is shown in Figure 3.

(6) $$\begin{array}{l} \text{RSH}+\text{1}/\text{4}{\text{O}}_{\text{2}}\begin{array}{c} \text{Catalyst}\\ \to \\ \text{lye} \end{array}\text{1}/\text{2 RSSR}+\text{1}/\text{2}{\text{H}}_{\text{2}}\text{O} \end{array}$$

### Dearomatization Experiment

The stock hydrocarbons was treated further in a hydrodearomatization experiment. The hydrogenation of aromatic hydrocarbon compounds is reversible (Xie, 2014). The equation is as follows:

(7) $$\begin{array}{l} A+n/2H_{2}=AH \end{array}$$

Where A is the aromatic hydrocarbon compound and AH is the product of hydrogenation, i.e., cycloalkane.

The equilibrium concentration of the aromatic hydrocarbon compounds is expressed under the following conditions, namely, that the liquid-phase activity factor of the aromatic hydrocarbon compound A and the cycloalkane AH are equal to the fugacity, the hydrogen activity coefficient to the total pressure ratio is 1, and the hydrogen fugacity to the total pressure ratio is 1.

(8) $$\begin{array}{l} \frac{y_{A}}{y_{A}+y_{AH}}=\frac{1}{1+K_{a}\times {\left(P_{H_{2}}\right)}^{n}} \end{array}$$

Where *y*_*A*_ is the aromatic hydrocarbon mole fraction, *y*_*AH*_ is the cycloalkane mole fraction, *K*_*A*_ is the equilibrium constant, and *P*_*H*_2__ is the hydrogen differential pressure.

Higher pressure is beneficial to decreasing the equilibrium concentration of aromatic hydrocarbon and increasing its conversion rate. In the process of aromatic hydrocarbon hydrogenation, 63–71 A/kJ·mol^−1^ heat is released continuously. The equilibrium constant *K*_a_ decreases, and the equilibrium conversion rate decreases with the increase in reaction temperature. Therefore, a lower temperature is more conducive to the reaction, so that benzene and toluene are converted maximally into saturated cyclohexane and saturated methylcyclohexane (Wang et al., 2013; Wang, 2015; Chen et al., 2020).

Through Table 7, as the hydrogenation kinetics of polycyclic aromatic hydrocarbons (PAHs) follow the “cyclic hydrogenation principle,” the hydrogenation rate of the PAHs decreases in turn, with the first cyclic hydrogenation being the fastest and the last aromatic hydrogenation being slower than that of the monocyclic aromatic hydrocarbons. Consequently, there are more monocyclic aromatics and monocyclic cycloalkane in hydrocracking products. It can be concluded that benzene and hydrogen produce cyclohexane, which can improve the yield of aromatic hydrocarbon hydrogenation effectively under low-temperature and high-pressure conditions.

**Table 7:** The aromatics hydrogenation kinetic parameters under different reaction conditions.

**Hydrocarbon chemical compound**	**Quasi-reaction rate constant/(min**^****−1****^**)**			**Activating energy/(A/kJ·mol^**−1**^)**	**Ln (A/kJ·mol^**−1**^)**
	**340^**°**^C**	**360^**°**^C**	**380^**°**^C**		
Alkyl benzene	0.33 ± 0.01	0.53 ± 0.06	0.66 ± 0.09	58.00 ± 13.00	10.10 ± 2.50
Asphalt base benzene	0.32 ± 0.03	0.46 ± 0.11	0.64 ± 0.09	45.00 ± 9.00	7.50 ± 1.60
Bicyclo alkyl benzene	0.25 ± 0.06	0.34 ± 0.15	0.39 ± 0.09	40.00 ± 10.00	6.20 ± 1.90
Fragrant carbon	0.31 ± 0.07	0.45 ± 0.09	0.51 ± 0.11	44.00 ± 11.00	7.50 ± 1.20

The comparison and evaluation results of the density, viscosity, and flash point of various synthetic fluids after desulfurization and dearomatization are shown in Table 8.

**Table 8:** The performance of the base fluid data sheet.

**Properties**	**Gas oil**	**NSF**	**Bio-oil**
ρ/(kg·m^−3^)	851	780	790
Flash point/°C	111	134	134
Flow point/°C	15	−55	−30
Aromatic hydrocarbon content/quality %	18.2	0.3	0.5
Sulfur content/%	<0.1	<0.1	<0.1
Viscosity /(mPa·s)	4.0	1.5	1.8
LC50 value/(mg·l^−1^ WSF)	>1,000,000	>1,000,000	>1,000,000

## Results

Our newly prepared NSF is composed mainly of C_12_-C_22_ hydrocarbons with a low impurity content, low specific gravity, low water content, easy volatilization, and easy degradation.

### Number of Carbon Atoms in NSF

**Figure 4:**
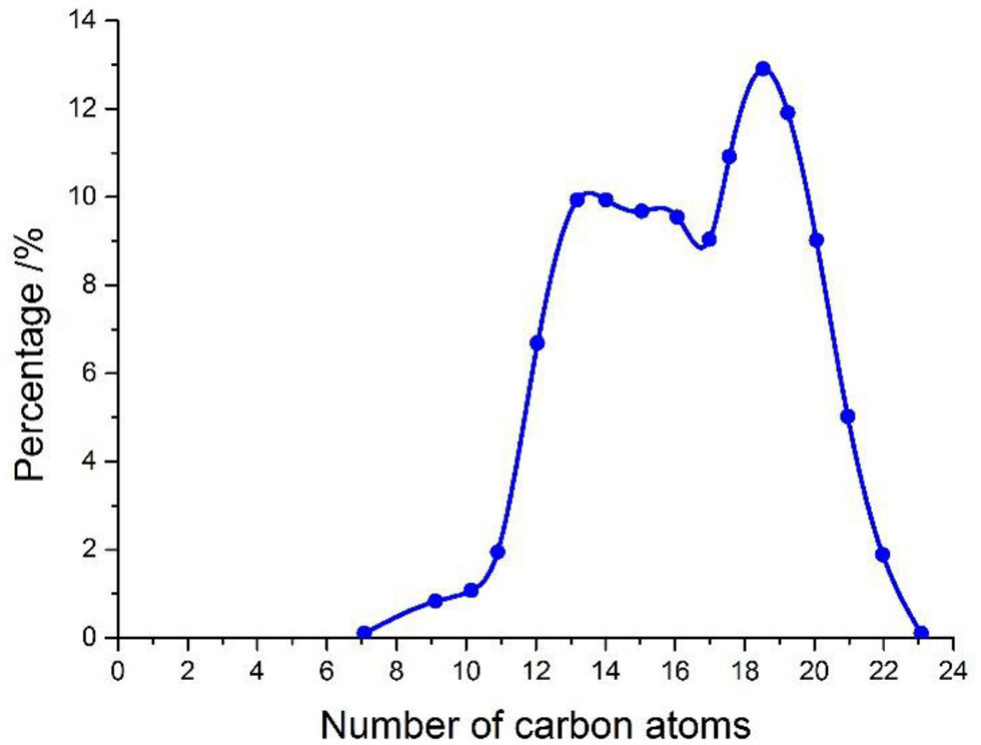
Number of carbon atoms in NSF under ambient condition.

As shown in Figure 4, the C_12_-C_22_ content of carboatomic numberin NSF is more than 90%.

### KV of NSF

**Figure 5:**
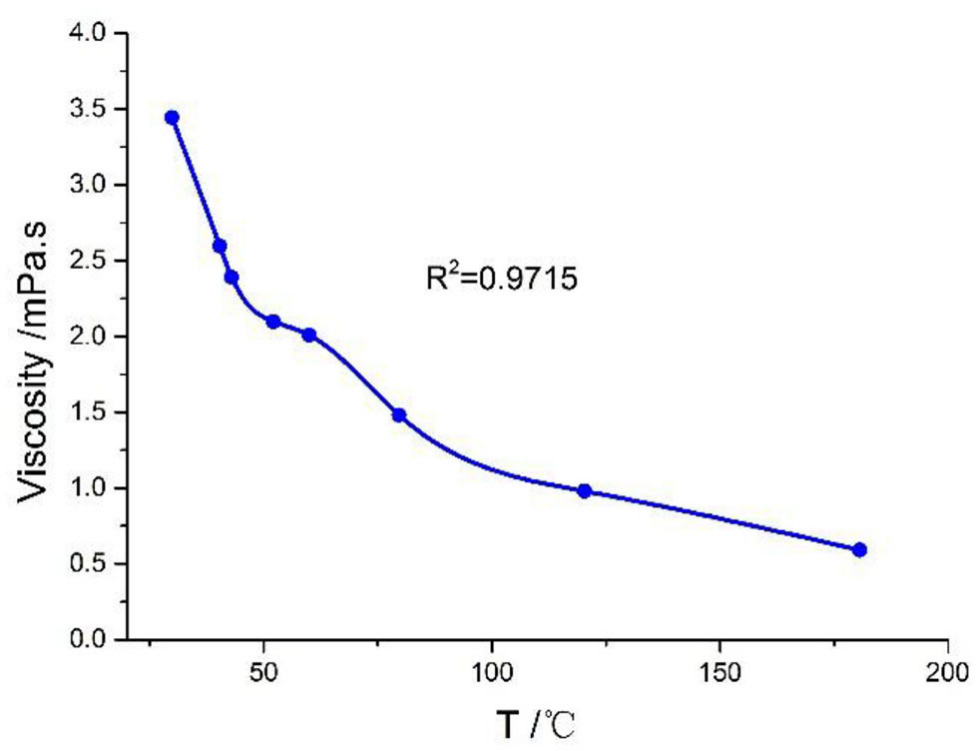
Viscosity with temperature change curve under atmospheric pressure.

As shown in Figure 5, the kinematic viscosity (KV) of NSF is low, and the viscosity of NSF decreases with the increase in temperature.

### Density of NSF

**Figure 6:**
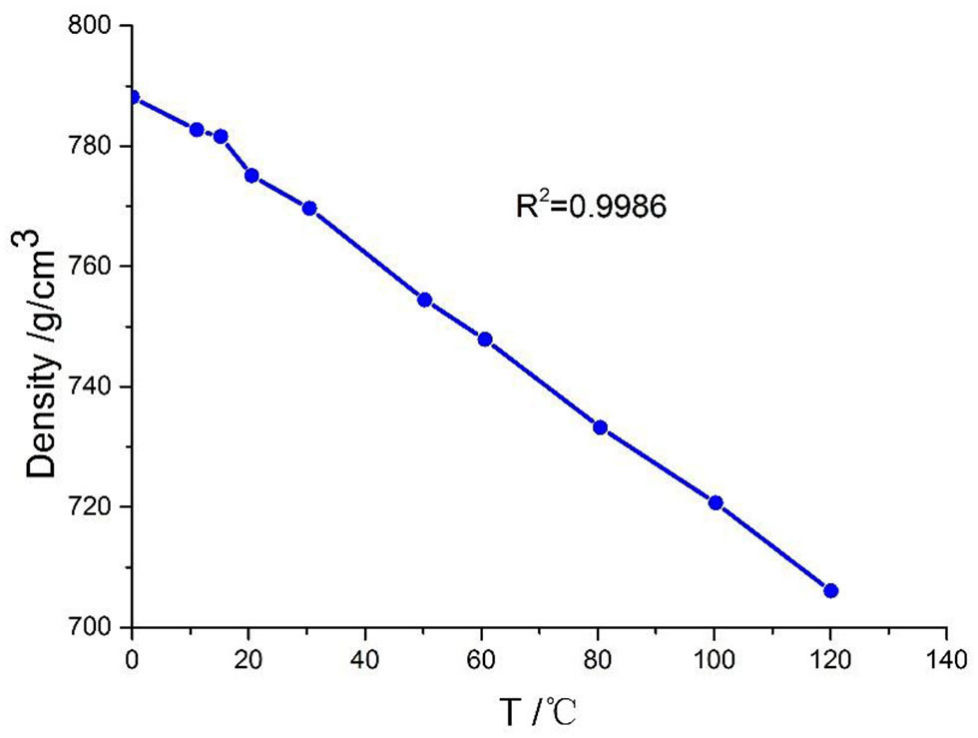
Density changing with temperature curve under atmospheric pressure.

As shown in Figure 6, the density of NSF is affected significantly by temperature, e.g., with an increase in the temperature, the density of NSF decreases.

### Compressibility of NSF

**Figure 7:**
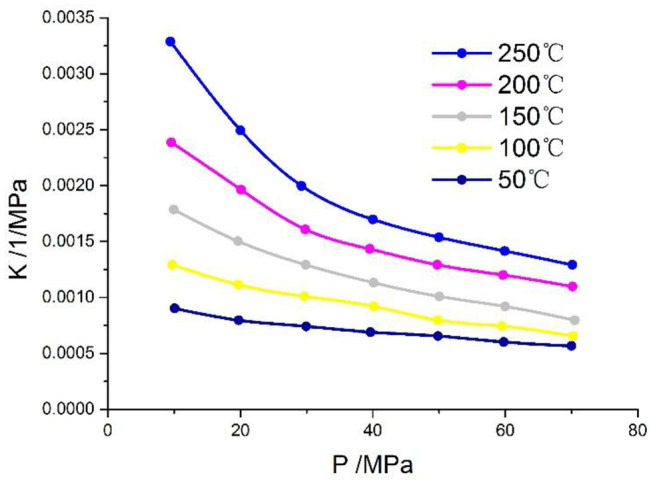
Compression performance change under different temperature and pressure.

As shown in Figure 7, as the temperature increases, the compressibility of NSF increases. And the higher the pressure, the smaller the diffenerce between compressions.

## Discussion

To ensure that the performance of the synthetic-based drilling fluid with the designed synthetic fluid meets the drilling requirements, a synthetic based drilling fluid is formulated with additives, such as viscosifier, emulsifier, and fluid loss control agent, etc. The formula is as follows:

250 mL synthetic fluid + 20% brine (5% CaCl_2_) + 3% emulsifier + 3% viscosifier + 2% wetting agent (WETA-2) + 3% acryl resin + lime + barite.

### Rheological Property of Synthetic-Based Drilling Fluid

Laboratory experiments on the rheological property of the drilling fluid with different densities are conducted. Our experimental results showed that the synthetic-based drilling fluid has good rheological property and a low filtration rate.

As shown in Table 9, the synthetic-based drilling fluids with different densities have good rheological property and low medium pressure filtration, which can improve the rate of penetration effectively.

**Table 9:** Performance of synthetic-based drilling fluid under different density.

**ρ/(g/cm^**3**^)**	**Conditions**	**FL API/mL**	**Gel Pa/Pa**	**AV/(mPa·s)**	**PV/(mPa·s)**	**YP/Pa**	**Φ6/Φ3**
1.32	Before aging	0.6	2.0/8.0	25	23	2	3/2
	After aging		9.0/12.0	32	21	11	23/21
1.45	Before aging	0.9	1.5/3.0	28	26	2	5/3
	After aging		7.0/15.0	33	16	17	22/17
1.56	Before aging	1.3	2.0/3.0	30	27	3	5/4
	After aging		13.0/17.0	39	20	19	27/24
1.68	Before aging	1.5	2.5/3.5	35	27	8	6/4
	After aging		11.0/15.0	43	22	21	29/27
2.00	Before aging	1.7	9.0/13.0	71	56	15	18/15
	After aging		12.0/15.0	75	50	25	29/24

*Aging for 16 h at 150°C and the testing temperature is 50°C*.

### Sag Stability of Synthetic-Based Drilling Fluid

Synthetic-based drilling fluid, with a density of 1.50 g/cm^3^ was aged at 150°C for 16 h, and the density difference between the upper and lower parts of the drilling fluid in the beaker was measured after holding for 24 h. The results showed that the density of the upper drilling fluid was 1.49 g/cm^3^ and that of the lower was 1.52 g/cm^3^, indicating that the synthetic fluid had not precipitated. Accordingly, the synthetic-based drilling fluid has excellent suspension property.

As shown in Table 10, under a simulated down hole temperature condition, the viscosity and shearing force of the drilling fluid increased with an increase in the resting time. The drilling fluid showed good shear thinning property, strong suspension force, and solid carrying capacity, and could meet various operating requirements under pump-off conditions.

**Table 10:** ρ = 1.50 g/cm^3^ density of drilling fluid performance comparison before and after high temperature standing.

**Conditions**	**L/mL**	**V/s**	**Gel Pa/Pa**	**AV/(mPa·s)**	**PV/(mPa·s)**	**YP/Pa**	**Φ6/Φ3**	***N***	***K***
Before standing	1	51	7/9	46	38	8	11/9	0.77	163.24
After standing	2	57	19/21	81	53	28	41/39	0.57	329.51

*Aging for 16 h at 150°C and the testing temperature is 50°C, standing for 24 h*.

### Electric Stability of Synthetic-Based Drilling Fluid

Synthetic-based drilling fluids with different densities were used to determine the electric stability (Kirsner et al., 2005; Yi et al., 2008), as shown in Table 11. The demulsification voltage of these drilling fluids at different densities was more than 2,000 V, indicating that the system has good electric stability.

**Table 11:** Different drilling fluid density electric stability.

**ρ/(g/cm^**3**^)**	**Based fluid**	**80^**°**^C VB ES/V**	**150^**°**^C VB ES/V**
1.3	NSF	>2,000	>2,000
1.4	NSF	>2,000	>2,000
1.5	NSF	>2,000	>2,000

*Aging for 16 h at 80°C, aging for 16 h at 150°C, and the testing temperature is 50°C*.

As shown in Table 11, the densities of the drilling fluid that we choose are 1.3, 1.4, and 1.5 g/cm^3^ and no matter what the density condition is, the demulsification voltage under 80 and 150°C is always higher than 2,000 V, which shows a good electric stability.

### Reservoir Protection Performance of Synthetic-Based Drilling Fluid System

The core of Rang 53 Block was selected to evaluate the performance of synthetic-based drilling fluid through laboratory experiments. The procedure s are as follows: after choosing the core, first, determining the permeability of rock samples with air; next, vacuuming the rock sample and saturate it with formation water, and displacing it with kerosene under until the formation reaches irreducible water under the pressure difference between confining pressure and inlet pressure of 1.5–2.0 MPa, then measuring the positive permeability (K_0_). Finally, pumping the different drilling fluids from the negative part, and then measuring the positive permeability (K_1_) with kerosene after damaging. The ratio of K_1_ to K_0_ is the permeability recovery values. All of the above process is implemented under ambient temperature for about 120 min. The drilling fluid affected the reservoir in both solid and liquid phases, i.e., the invasion of the solid particles and the invasion of the filtrate (Zhang et al., 2017; Zhang, 2018). The permeability recovery value is an index for evaluating the degree of reservoir damage. Different drilling fluid systems were selected to determine the permeability recovery values. The experimental results under atmospheric conditionare shown in Table 12.

**Table 12:** Different pollution system core permeability recovery value.

**Drilling fluid system**	**Core**	**d/mm**	**L/mm**	**K/md**	**K_**0**_/md**	**K_**1**_/md**	**K_**1**_/K_**0**_/%**
Synthetic-based drilling fluid	1#	25.35	31.27	140.60	50.15	49.19	95.20
Gas-based drilling fluid	2#	25.60	33.36	162.27	58.53	49.76	90.25
Bio-oil based drilling fluid	3#	25.42	33.64	180.53	53.47	49.77	93.08

As shown, the permeability recovery value of a conventional drilling fluid system is kind of low, and the damage to the formation is significant, i.e., it is blocked and polluted easily. However, the core permeability recovery value of the synthetic-based drilling fluid system is more than 95%, the filtration rate is low, and reservoir protection effect is good.

### Dynamic Filtration Evaluation of Synthetic-Based Drilling Fluid

Dynamic filtration experiments with the synthetic-based drilling fluid, gas-based drilling fluid, and bio-oil based drilling fluid are carried out. The procedures are as follows: drilling fluid is poured into the mud tank of the dynamic filtration simulator and heated. When the temperature reaches 5–10°C below the experimental temperature, the motor is turned on to the required speed. When the temperature is constant, the filtration volume is recorded until the dynamic filtration is in equilibrium. Then, the experiment is stopped (Zhang et al., 2016; Gong et al., 2017). The results are shown in Table 13.

**Table 13:** The dynamic filtration experiment results.

**Number**	**Types**	**2 h accumulated filtration rate/mL**
1	Bio-oil based drilling fluid	0.5
2	Gas-based drilling fluid	1.0
3	Synthetic-based drilling fluid	0.3

*Temperature is 120°C, shear rate is 0.77 m/s, differential pressure is 3.5 MPa, and confinement pressure is 6.0 MPa*.

As shown in the Table 13, when the drilling fluid is immersed in the formation too long, less dynamic filtration of the synthetic-based drilling fluid occurs and the filtrate entering the formation is insignificant, which is beneficial to wellbore stability.

## Conclusions

(1) Designed synthetic fluid is obtained from stock hydrocarbons by fractionation, hydrogenation, desulfurization, dearomatization, cooling, and other processes. NSF is composed mainly of C_12_-C_22_ hydrocarbons, with low impurity contents, small specific gravity, and easy degradability. The LC50 value of treated synthetic fluid is >1,000,000 mg/L WSF, and its toxicity is <1/10 of that of diesel. The flash point of NSF is above 134°C, the safety is high, and the aromatic hydrocarbon content of synthetic fluid is therefore significantly lower than is that of other synthetic fluid, such as bio-oil.

(2) We evaluated NSF systematically in the laboratory, with the results indicating that the properties of the designed NSF being clearly superior to those of other synthetic fluid. It is the preferred synthetic fluid for preparing environmentally friendly drilling fluid systems because of its high permeability recovery value which is 95.2%, excellent suspension property which with the upper drilling fluid was 1.49 g/cm^3^ and that of the lower was 1.52 g/cm^3^, minor dynamic filtration which is 0.3 mL in 2 h, and insignificant pollution of the reservoir.

(3) The prepared NSF is a quality product that does not cause pollution to the environment. This new synthetic-based drilling fluid is not only environmentally friendly but also its performance is superior and economic, because it can improve the rate of penetration without inducing borehole problems. We have therefore achieved our aim of preparing a kind of low-cost, high-quality, non-toxic synthetic fluid, of which the aromatic hydrocarbon and sulfur contents meet international standards. Using this synthetic-based drilling fluid therefore facilitates environment protection drilling.

## Data Availability Statement

All datasets generated for this study are included in the article/supplementary material.

## Author Contributions

YS: conceptualization, resources, and supervision. XZ and JZ: methodology. XZ and DS: validation and data curation. YS and JZ: formal analysis. XZ: investigation. YS and XZ: writing—original draft preparation and visualization. YS, XZ, and JZ: writing—review and editing. All authors contributed to the article and approved the submitted version.

## Conflict of Interest

The authors declare that the research was conducted in the absence of any commercial or financial relationships that could be construed as a potential conflict of interest.
